# Assessment of nurse’s perceived just culture: a cross-sectional study

**DOI:** 10.1186/s12912-023-01478-4

**Published:** 2023-10-03

**Authors:** Kenneth Jun Logroño, Badriya Abdulla Al-Lenjawi, Kalpana Singh, Albara Alomari

**Affiliations:** 1 Medical In-Patient Nursing, Hamad Medical Corporation, Doha, Qatar; 2 Nursing and Midwifery Research Department, Hamad Medical Corporation, Doha, Qatar; 3 College of Health Sciences, University of Doha for Science and Technology, Doha, Qatar

**Keywords:** Errors, Just culture, Patient safety, Safety culture, Quality and Safety

## Abstract

**Background:**

The non-punitive approach to error investigation in most safety culture surveys have been relatively low. Most of the current patient safety culture measurement tools also lack the ability to directly gauge concepts important to a just culture (i.e. perceptions of fairness and trust). The purpose of this study is to assess nurses’ perceptions of the six just culture dimensions using the validated Just Culture Assessment Tool (JCAT).

**Methods:**

This descriptive, cross-sectional study was conducted between November and December 2020. Data from 212 staff nurses in a large referral hospital in Qatar were collected. A validated, self-reported survey called the JCAT was used to assess the perception of the just culture dimensions including feedback and communication, openness of communication, balance, quality of event reporting process, continuous improvement, and trust.

**Results:**

The study revealed that the overall positive perception score of just culture was (75.44%). The strength areas of the just culture were “continuous improvement” dimension (88.44%), “quality of events reporting process” (86.04%), followed by “feedback and communication” (80.19%), and “openness of communication” (77.55%) The dimensions such as “trust” (68.30%) and “balance” (52.55%) had a lower positive perception rates.

**Conclusion:**

A strong and effective just culture is a cornerstone of any organization, particularly when it comes to ensuring safety. It places paramount importance on encouraging voluntary error reporting and establishing a robust feedback system to address safety-related events promptly. It also recognizes that errors present valuable opportunities for continuous improvement. Just culture is more than just a no-blame practice. By prioritizing accountability and responsibility among front-line workers, a just culture fosters a sense of ownership and a commitment to improve safety, rather than assigning blame.

## Background

The underreporting of medical errors is one of the most significant challenges to improve patient safety in healthcare [1, 2]. The Institute for Safe Medication Practices (ISMP) defined “errors” as an inevitable, unpredictable, and unintentional failure caused by human behavior and system failures [3]. Although most errors do not result from the reckless behavior but from faulty systems and processes [4], human factors (i.e. overwork, fatigue, memory lapses, staffing, distractions) always had been a contributing factor that challenged healthcare systems to improve patient safety [1, 2].

Front-line health professionals are reluctant to report errors for fear of punishment or blame [1, 5–7] and lack of belief that reporting will lead to improvement [6]. This fear might be a result of negative outcomes such as a malpractice lawsuit, losing patients’ trust, emotional reactions from patients and their families, or losing one’s job [7]. Although nurses have competing work demands and are forced to improvise and develop workarounds [8], nurses can have a significant impact on reducing errors due to their proximity to patients.

It has been established that the fear of repercussions can be eliminated by adopting a “just culture” where front-line staff are empowered that errors in any safety-related events are opportunities for continuous improvement [1]. Creating a culture characterized by voluntary error reporting [4–6] and learning from mistakes is necessary in building a just culture [9]. Just culture is a concept first introduced in the aviation industry in the 1980s [10]. As originally defined by James Reason, it is “a collective understanding between blameless and blameworthy actions” [10]. Just culture also helps organizations determine if an individual’s behavior represents a human error, at-risk behavior, or reckless behavior [11, 12]. For example, reckless behavior or the conscious disregard of a substantial and justifiable risk of harm should be differentiated from at-risk behaviors and human error [3]. More importantly, just culture is not about finding fault, it is about managing risk [11]. It supports disciplinary actions against individuals or organizations who engage in reckless behavior or willfully violate policies and the standards of care [11]. That means, ensuring a balanced accountability for both individuals and the organization responsible for designing and improving the systems in the workplace [6, 13].

Healthcare organizations should develop just culture in all levels from leaders and managers to front-line workers [9, 14]. In just culture, front-line workers are not blamed or punished, but ensured fair investigations are in place [12], and after an incident, the question asked is, “What went wrong?” rather than “Who caused the problem?” [15]. In terms of reporting the errors, just culture supports the value of voluntary reporting to redesign faulty systems rather than focusing on individuals [5, 9]. It cultivates a strong safety practice by encouraging fair and just treatment for front-line health professionals involved in any safety-related events [9].

Just culture assessment, as compared in assessing the safety culture in general which is usually done by most organizations, is a practical and novel way to investigate how an organization develops a non-punitive approach to error investigation [9, 13]. A systematic review was conducted over 13 patient safety culture instruments and cited that some tools are not focus on specifically assessing the non-punitive approach to errors such as the Safety Attitudes Questionnaire (SAQ), and Safety Climate Questionnaire (SCQ) [16]. Although the Hospital Survey on Patient Safety Culture (HSOPSC) has questions addressing the blame and non-punitive response to errors, it still lacks the ability to directly gauge specific concepts important to a fair, just and no blame practice (i.e. perceptions of fairness and trust) [9, 16]. However, these tools are very efficient in providing a broader view of organizational safety culture. Another significant aspect is that just culture continues to exist as an aspect of a safety culture [9, 11], meaning that certain elements of the overall patient safety culture (i.e. staffing, handoffs and transitions, and job satisfaction), [16], are less intuitive to assess the non-punitive approach to error investigation [9]. Additionally, most of the safety culture survey instruments reviewed (i.e. HSOPSC, SCQ, SAQ), are generalist in their focus which did not reveal a valid and reliable tool that explicitly assesses just culture in healthcare organizations [9, 16], they were designed to address a broad array of safety culture issues [16].

The development of the Just Culture Assessment Tool (JCAT) is a direct response to the practical needs of the organization to effectively distinguish between overall patient safety culture, and a just culture for patient safety [9, 17]. The JCAT also specifically allows measurement of different aspects of just culture such as feedback and openness of communication, balance accountability, quality of the reporting system, continuous improvement from errors, and trust from the management [9]. Although there were overlapping elements between survey tools of just culture and safety culture, the latter still lacks the ability to assess front-line workers; beliefs, attitudes, and experiences regarding the organization’s response to error [9]. This paper also addressed the dimensions that were important to overcome underreporting of errors such as the impact of electronic incident reporting system, and a balance of accountability and trust towards the management in handling error investigations which were not present in most safety culture surveys.

Furthermore, a research study conducted on the assessment of perceived safety culture of nurses in a large referral hospital in Qatar [18], showed that the actual error reporting was only 34.00%. Majority of nurses (76.00%) felt like their mistakes are held against them, and 66.00% of them feel like an individual is being incriminated when an incident report is filed [18, 19]. Conversely, the database report of the Agency for Healthcare Research and Quality (AHRQ), showed that the non-punitive response to error dimension has consistently been the lowest-scoring category [18–20]. The paucity of empirical evidence demonstrates that further research is required to identify causes of relatively poor just culture practice and identify effective strategies for establishing or maintaining a just culture that will enhance patient outcomes and healthcare safety [2].

Moreover, gaps in existing research on safety culture were identified in that there is a lack of studies about just culture that have been conducted [9, 13, 21, 22]; and there have been limited research published in exploring the perceptual dimensions of just culture, especially in the Middle East region [22, 23].

## Methods

### Study aim

This study aims to assess the perceived just culture among nurses in Qatar in terms of Feedback and Communication; Openness of Communication; Balance; Quality of Events Reporting Process; Continuous Improvement; and Trust.

### Study setting

The study was conducted in the Medical and Surgical In-Patient Departments in a large tertiary referral hospital in Doha, Qatar. The departments had a total of 727 staff nurses, offering high-level specialized clinical services and care. The medical and surgical departments were specifically chosen as the focus of this study, as they encompass a wide range of nursing backgrounds that effectively mirror the diverse nursing workforce in Qatar. The in-patient departments were also chosen as they were accessible for the researchers involved and the only approved study setting by the hospital management.

### Study design and sampling

A descriptive, cross-sectional research design was used. All staff nurses in medical and surgical in-patient wards were invited to participate in this study. The study utilized a simple random sampling technique through a Research Randomizer [24] from the general list of staff nurses in both Medical and Surgical In-Patient Departments. The standard deviation used in the formula was taken from the “continuous improvement” dimension in the prior study which is 0.70 [9], to arrive at the most practical sample size. Hence, the computed sample size of 212 nurses was needed in the study with ± 8% level of precision and 95% confidence interval.

### Data instrument

The validated JCAT developed by Petschonek et al. was adapted and utilized [7]. JCAT consisted of 27 questions and had six dimensions which included 1.) Feedback and Communication (3 items); 2.) Openness of Communication (5 items); 3.) Balance (5 items); 4.) Quality of Event Reporting Process (5 items); 5.) Continuous Improvement (4 items); and 6.) Trust (5 items). The definitions of each dimension are summarized in Table 1, where it is used to discuss the concepts of just culture perceptions which are composed of six (6) distinct dimensions [9].

**Table 1:** Just culture dimensions and its definitions

Dimension	Definition
Feedback and Communication about Events	One’s beliefs regarding whether the organization does an effective job of sharing event information about the events and the outcome of evaluating events
Openness of Communication	The willingness of individuals to communicate event information upwards to supervisors and hospital administrators e.g., willingness to reveal events, share events information, and to make suggestions for improvement within the unit or the organization
Balance	One’s perceptions of fair treatment within the hospital as it relates to errors, error reporting, and its systems approach to medical error
Quality of the Event Reporting Process	One’s perceived quality of the event reporting system (which includes the process of entering reports and the ability to follow up on these reports), whether employees are given time to report, and to what extent the employees believe the reporting system is monitored and maintained
Continuous Improvement	One’s belief that the organization demonstrates a goal of continuous improvement, characterized by a willingness to learn from events and make improvements to the hospital system
Trust	The extent to which individuals trust the organization, their supervisors, and their co-workers

The development of the JCAT is a direct response to the measurement and interpretation of different aspects of just culture. The JCAT is the first and the only questionnaire to measure various aspects of a just culture for patient safety [9]. The previous study which involved the development of the JCAT presented evidences of the validity and reliability of the tool [8, 9]. Confirmatory factor analysis was used to test the internal structure of the tool and reliability analyses were conducted on the subscales [9], where a Cronbach’s alpha reliability scores for the subscales were positive, with each dimension being above 0.70 [9].

The survey consisted of three (3) parts: 1.) consent form, 2.) the nurses’ demographic profile and 3.) survey questions on just culture with a 7-point Likert scale (Strongly Agree = 1 to Strongly Disagree = 7). It was administered online through a Q-survey program in an English version and took 10–15 min to complete. The questionnaire was reviewed prior to distribution to ensure that the terms were aligned with the terms used by the nurses. Modifications included changing and adding of some terms to facilitate better understanding on the questions and didn’t need a construct re-validation. Some modifications on the JCAT included changing terms that are not familiar to most of the staff nurses which included:

- The terms “medication errors”, “near misses”, “accidents” and “adverse incidents” were used to specify the term “events”, the item, “I do not know about events like any medication errors, any near misses, any accidents and any adverse incidents that happen in our unit.”
- The head nurse and charge nurses were added to the term “supervisors”, and the item, “I feel uncomfortable discussing events (like medication errors, near misses, accidents and adverse events) with supervisors, head nurses or charge nurses.”
- The term Occurrences, Variances and Accidents (OVA) was added with “events reporting”, and the item, “The event reporting system (or OVA system) is easy and friendly to use.”
- The term “tattle” was changed to “gossip” or “talk against on each other”, and the item, “Staff members use event reporting to [tattle] gossip or talk against on each other.”

### Data management and analysis

Data from the Q-survey were exported. The data was cleaned and prepared using Microsoft Excel, while STATA 17.0 was utilized for the actual analysis. Descriptive statistics, such as frequency, percentage, averages, and standard deviation for scale items, were used to analyze the data.

Descriptive statistics was used to assess the demographic profile. For analysis of JCAT, the number of positive responses was calculated for positively worded items as well as for reversely worded items. Additionally, a dimension-level response rate was calculated by getting the total number of responses in the respective consolidated levels of agreement (agree, neutral, disagree), and dividing it by the product of overall total number of surveyed nurses and total number of items in the dimension. There are three question items in the “feedback and communication” dimension, with a minimum score of 3 and a maximum score of 21. There are five item questions with a minimum score of 5 and a maximum score of 35 on the dimensions of “openness of communication”, “balance”, “quality of event reporting”, and “trust”. The “continuous improvement” dimension has four questions, each with a minimum of 4 points and a maximum of 28.

### Ethical consideration

This study was conducted in accordance with the Declaration of Helsinki on ethical standards and in full compliance with all the relevant sections of the Rules and Regulations for Research at Hamad Medical Corporation and the Medical Research Center. The study was approved by the Ethics Review Committee of the Medical Research Center to be conducted under the protocol no. MRC-01–20-962 last November 4, 2020.

The participants were informed of their rights to withdraw from participation at any time. Participation was voluntary. Informed consent was provided by the participants prior to their participation. The survey was anonymous, and confidentiality of the information was assured.

## Results

### Demographic characteristics

Of only 257 responses from the staff nurses, forty-five responses were eliminated due to incomplete data (i.e., failure to give consent, missing responses, and skipped some question items). After the data cleaning, a total number of 212 responses were analyzed. The demographic profile of the subjects is summarized in Table 2.

**Table 2:** Demographic profile of nurses (*n* = 212)

	**n (%)**	**Mean (SD)**	**Range**
**Age**		29.73 (6.44)	25–43
**Gender**			
**Female**	157 (74.54%)		
**Male**	55 (25.00%)		
**Nationality**			
**Asian** (Filipino, Indian, Indonesian)	197 (93.00%)		
**Middle Eastern** (Jordanian, Egyptian, Sudanese, Qatari, Yemeni)	15 (7.00%)		

The demographic characteristics included age, gender, and nationality. The mean age of the nurse participants was 29.73 ± 6.44 years. Almost 65.2% participants were young to middle-aged adult nurses and three fourth of staff nurses (74.54%) were *female*. Around 93.00% participants were Asians including Filipino, Indian, and Indonesian; and 7.00% were Middle eastern including Jordanian, Egyptian, Sudanese, Qatari, and Yemeni.

### Just culture perception

This study assessed the nurses’ perceptions of patient safety culture through a JCAT. The survey measured just culture under six (6) dimensions, as described above. The consolidated response rates for each dimension are summarized in Table 3 above:

**Table 3:** Consolidated response rates for just culture dimensions (*n* = 212)

Just Culture Dimension	**No. of Items**	Agree (%)	Neutral (%)	Disagree (%)
Feedback and Communication	**3**	80.19%	3.46%	16.35%
Openness of Communication	**5**	77.55%	5.57%	16.89%
Balance	**5**	52.55%	7.83%	39.62%
Quality of Events Reporting Process	**5**	86.04%	4.72%	9.25%
Continuous Improvement	**4**	88.44%	4.83%	6.72%
Trust	**5**	68.30%	8.96%	22.74%

The positive response rate of > 75.00% is considered to be the strength areas of any safety culture survey dimensions [25], such strength areas of the just culture were “continuous improvement” dimension (88.44%), and “quality of events reporting process” dimension came second, with 86.04% positive response rate. These are followed by “feedback and communication”, and “openness of communication” dimensions with positive response rates of 80.19% and 77.55%. The two lowest positive response rates were for “trust” (68.30%) and “balance” (52.55%), which needs improvement. It can also be observed that despite having high positive response rates, negative response rates are consistently higher than the neutral scores. The observations for each just cultural dimension, together with the detailed consolidated response rates for each item can also be observed that despite having high positive response rates, negative response rates are consistently higher than the neutral scores (data not shown).

**Fig. 1:**
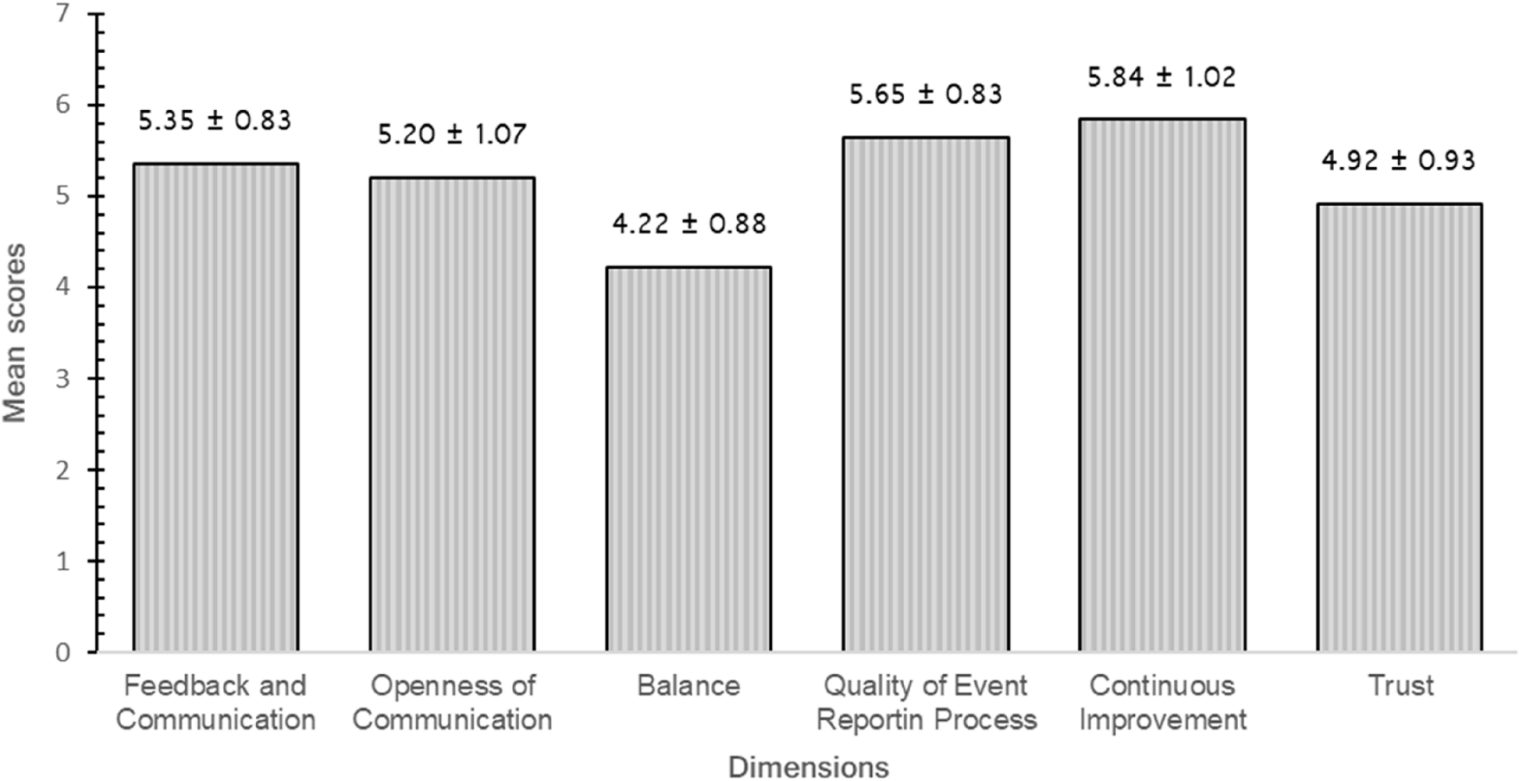
Average scores of six just culture dimensions (*n* = 212)

A systematic review study of on safety culture survey, considered perception as positive if the dimension had a mean score > 4.00 on a 7-point Likert scale, i.e., “strongly agree,” “agree,” or “somewhat agree” [26]. The extracted data were synthesized in a simple manner illustrated in Fig. 1.

The highest-rated just culture dimensions were “Continuous Improvement” (mean ± SD = 5.84 ± 1.02), “Quality of Events Reporting Process” (mean ± SD = 5.65 ± 0.83), “Feedback and Communication” (mean ± SD = 5.84 ± 1.02), and “Openness of Communication” (mean ± SD = 5.20 ± 1.07). The lowest rated just culture dimensions were “Trust” (mean ± SD = 4.92 ± 0.93) and “Balance” (mean ± SD = 4.22 ± 0.88). Though “trust” and balance” dimensions receiving the lowest scores, the results were still high when compared to similar just culture studies [9], and overall, there is a positive perception of just culture among nurses (mean = 5.20).

## Discussion

This unique study sought to understand the perception of nurses about the just culture. This is the first major study addressing just culture in a large referral hospital in Qatar. The nurses reported that creating a just, balanced and learning environment is more critical in managing errors than a punishment culture. They also reported that the quality of the reporting process, feedback, and openness of communication after committing errors are factors that may encourage nurses to report the incident.

The study revealed that the overall positive perception score of just culture was (75.44%). This is showing that the respondents are generally feeling positive about the current practice and they are feeling safe to report the incident without the “fear” of consequences. However, the result of the current research is contrary to a previous quantitative, cross-sectional study that was conducted among nurses in different referral hospitals in Qatar where authors found out that a non-punitive approach to errors resulted in a low positive perception score of only 23.00% [18, 19]. As was pointed out in the introduction to this paper, the safety culture tools lack the ability to gauge specific concepts in just culture like perceptions of fairness and trust which may contribute to the consistently low scores [9, 16]. Such approaches to these tools, however, have failed to address critical assessments of just culture, more than just a blame-free practice.

Results of this study were useful enough to validate that a supportive workplace where giving feedback about errors is common, and that nurses are more likely to disclose medication errors [27]. This can be illustrated briefly in a study which explored the relationship between characteristics of the nursing practice environment and rates of medication errors in acute care hospitals [28]. The authors found that a supportive culture, where there is a feeling of safety, was significantly associated with the prevention of medication errors [27].

The “continuous improvement” dimension, which represents the staff perception that errors can be learning opportunities to drive improvement rather than constraints [9], has the highest positive perception score of 88.44%. In contrast to a previous descriptive and analytical study, using a questionnaire, conducted among 100 nurses, authors found out that non-disclosure to medication errors were related to lack of learning [29]. The authors recommended that managers should reinforce the culture of the importance of complete reporting and create an opportunity for the nurses to learn from these reports. This related to a hypothetical model called “a map of learning”, highlighting the importance of working collaboratively to learn from incidence reporting rather than punishing the staff [30]. Having an opportunity to receive information and learn from incidence can help in learning and provides everyone with the opportunity to see different perspectives, which may influence or fundamentally change the way participants analyze the knowledge [31]. Learning from incident reporting is a continuous quality improvement effort [31], which is a critical aspect in just culture practice. This can be achieved by educating nurses about all aspects of the reporting process [31]. If nurses have more knowledge about why they must report and how the administration of the organization handles these reports after they are received, they will be more voluntary and compliant [31].

The “quality of event reporting” was the second most important factor among nurses. There was a strong correlation between the perception of a blame-free work environment and the number of electronic incident reports received because of the easy process of reporting [32]. This is consistent with the result of the recent literature where the electronic incident reporting system is essential in achieving a quality event reporting process [8, 33]. The electronic incident reporting system is currently being utilized by the healthcare system in Qatar. It can simplify the process of documentation of event reporting making it more accessible, easier to use, and analyze data more efficiently.

Additionally, the anonymity or de-identification of entering reports also helps increase the compliance to voluntary error reporting, which contributes to the “culture of trust” [8, 32]. In a study aimed at clarifying the factors associated with reporting nursing errors (interviewing 115 clinical nurses and nurse managers), the nurses reported that they were afraid of losing their honor and dignity or being stigmatized [34]. Anonymous reporting option has been embedded in the incident reporting system in major hospitals in Qatar. It is recommended that the error reports should be de-identified, and the nurses involved in these reports should be unknown to other nurses [34]. De-identifying the reports also has the effect of making the reporting nurses feel as though their reputation is unharmed when an error occurs, which motivates them to encourage error reporting in furthering safety in their current practice [35].

Nurses in the current study were positive about “feedback” and the “openness of communication*”* dimensions. This only proves that communication about errors is an important aspect of safety culture [8, 13]. It is important to have two-way communication about the process [9], staff nurses must be willing to openly communicate about events and hospital leaders must be willing to provide feedback and updates about how that information is being used to improve patient safety. Regular reporting of error trends and system failures should be communicated to all staff nurses [4]. Patients and clinicians also benefit from disclosure of errors because it provides timely answers to questions about the incidents and reduces the need for lengthy litigation [33]. This is consistent with a previous qualitative study, using focus groups with nurses, aimed to identify medication error reporting beliefs [31]. Participants indicated that they would report medication errors more frequently if they received feedback, as they could learn from their mistakes and then improve their safety practice [31]. They concluded that this type of culture stimulates continuous quality improvements, which maintains a positive reporting culture. Effective and open communication between the management and nurses may empower them to report incidence and learn from them [36].

Some aspects of safety culture alone, however, are not sufficient to address safety behavior and the non-punitive approach to error investigation [9, 11]. Given this gap, the concept of just culture, a component of safety culture, evolved. Such dimensions like “balance “and “trust” are critical in understanding just culture for patient safety.

As stated earlier, a just culture is one in which nurses trust their organization to treat each incident as an opportunity to improve safety and feel they will be treated fairly if they are involved in any patient safety incident [9]. The “trust” dimension with 68.30% positive perception score confirmed that nurses were more open in discussing the events if they had trust and confidence in their supervisors [9]. However, self-reporting of errors has serious consequences [33], such as inhibiting openness to discuss errors because of blame [6], and punishment [6, 8]. In this study, 57.55% of nurses perceived that they were not blamed following any patient safety events. However, these results were not very encouraging as only half of the respondents agreed. This only proves that just culture is to maintain balance for fair and non-punitive approach to errors [9].

Comparison of the findings with those of other studies confirms that just culture isn’t just deliberately avoiding laying blame, rather a culture of balance accountability [8, 12]. The “balance” dimension is composed of both non-punitive treatment as well as individual’s accountability [9]. The “balance” dimension had the lowest positive perception score of only 52.55%. “Balance” is one’s perceptions of fair treatment within the hospital as it relates to errors, error reporting, and its systems approach to medical error [9]. Although most nurses in this study are not feeling blamed (57.55%), incident reports are used to tattle or talk about individuals involved in safety-related events (43.87%) which pulled the positive perception rate of “balance” dimension. The results showed similarities with many just culture research studies around the world [9, 37]. Tattling about the errors or any safety events increased anxiety among individuals as rumors circulate without clear information as to what is and isn’t a fact. It may also cause divisiveness among individuals without clear information on the incident. The increased negative gossip provokes an environment of low interpersonal trust [9, 37], and might cause work disruptions.

The definition of “balance” in this study also helps distinguish the difference between a no blame culture and just culture. A no blame culture gives people a false sense of their actions and mistakes have no impact on the patient and organization [12]. Just culture, on the other hand [9], assigns responsibility and accountability for the consequences of their actions [14]. Healthcare organizations cannot afford a blame-free culture and that some errors do warrant disciplinary action [12]. Finding a balance between the punishment and blamelessness is the ultimate goal of developing a just culture [11, 35]. Furthermore, Reason is credited for just culture’s inception as “a collective understanding of where the line should be drawn between blameless and blameworthy actions” [9, 10]. This is consistent with the previous research findings that a just culture cannot be a blame-free enterprise and that balanced accountability is significant in achieving a strong safety practice [8, 12].

## Limitations

‬‬‬‬‬‬‬‬‬‬‬‬‬‬‬‬‬‬‬‬‬‬‬‬‬‬‬‬‬‬‬‬‬‬‬‬‬‬‬‬‬‬‬‬‬‬‬‬‬‬‬‬‬‬‬‬‬‬‬‬‬‬‬‬‬‬‬‬‬‬‬‬‬‬‬‬‬‬‬‬‬‬‬‬‬‬‬‬‬‬‬‬‬‬‬‬‬‬‬‬‬‬‬‬‬‬‬‬‬‬‬‬‬‬‬‬‬‬‬‬‬‬‬‬‬‬‬‬‬‬‬‬‬‬‬‬‬‬‬‬‬‬‬‬‬‬‬‬‬‬‬‬‬‬‬‬‬‬‬‬‬‬‬‬‬‬‬‬‬‬‬‬‬‬‬‬‬‬‬‬‬‬‬‬‬‬‬‬‬‬‬‬‬‬‬‬‬‬‬‬‬‬‬‬‬‬‬‬‬‬‬‬‬‬‬‬‬‬‬‬‬‬‬‬‬‬‬‬‬‬‬‬‬‬‬‬‬‬‬‬‬‬‬‬‬‬‬‬‬‬‬‬‬‬‬‬‬‬‬‬‬‬‬‬‬‬‬‬‬‬‬‬‬‬‬‬‬‬‬‬‬‬‬‬‬‬‬‬‬‬‬‬‬‬‬‬‬‬‬‬‬‬‬‬‬‬‬‬‬‬‬‬‬‬‬‬‬‬‬‬‬‬‬‬‬‬‬‬‬‬‬‬‬‬‬‬‬‬‬‬‬‬‬‬‬‬‬‬‬‬‬‬‬‬‬‬‬‬‬‬‬‬‬‬‬‬‬‬‬‬‬‬‬‬‬‬‬‬‬‬‬‬‬‬‬‬‬‬‬‬‬‬‬‬‬‬‬‬‬‬‬‬‬‬‬‬‬‬‬‬‬‬‬‬‬‬‬‬‬‬‬‬‬‬‬‬‬‬‬‬‬‬‬‬‬‬‬‬‬‬‬‬‬‬‬‬‬‬‬‬‬‬‬‬‬‬‬‬‬‬‬‬‬‬‬‬‬‬‬‬‬‬‬‬‬‬‬‬‬‬‬‬‬‬‬‬‬‬‬‬‬‬‬‬‬‬‬‬‬‬‬‬‬‬‬‬‬‬‬‬‬‬‬‬‬‬‬‬‬‬‬‬‬‬‬‬‬‬‬‬‬‬‬‬‬‬‬‬‬‬‬‬‬‬‬‬‬‬‬‬‬‬‬‬‬‬‬‬‬‬‬‬‬‬‬‬‬‬‬‬‬‬‬‬‬‬‬‬‬‬‬‬‬‬‬‬‬‬‬‬‬‬‬‬‬‬‬‬‬‬‬‬‬‬‬‬‬‬‬‬‬‬‬‬‬‬‬‬‬‬‬‬‬‬‬‬‬‬‬‬‬‬‬‬‬‬‬‬‬‬‬‬‬‬‬‬‬‬‬‬‬‬‬‬‬‬‬‬‬‬‬‬‬‬‬‬‬‬‬‬‬‬‬‬‬‬‬‬‬‬‬‬‬‬‬‬‬‬‬‬‬‬‬‬‬‬‬‬‬‬‬‬‬‬‬‬‬‬‬‬‬‬‬‬‬‬‬‬‬‬‬‬‬‬‬‬‬‬‬‬‬‬‬‬‬‬‬‬‬‬‬‬‬‬‬‬‬‬‬‬‬‬‬‬‬‬‬‬‬‬‬‬‬‬‬‬‬‬‬‬‬‬‬‬‬‬‬‬‬‬‬‬‬‬‬‬‬‬‬‬‬‬‬‬‬‬‬‬‬‬‬‬‬‬‬‬‬‬‬‬‬‬‬‬‬‬‬‬‬‬‬‬‬‬‬‬‬‬‬‬‬‬‬‬‬‬‬‬‬‬‬‬‬‬‬‬‬‬‬‬‬‬‬‬‬‬‬‬‬‬‬‬‬‬‬‬‬‬‬‬‬‬‬‬‬‬‬‬‬‬‬‬‬‬‬‬‬‬‬‬‬‬‬‬‬‬‬‬‬‬‬‬‬‬‬‬‬‬‬‬‬‬‬‬‬‬‬‬‬‬‬‬‬‬‬‬‬‬‬‬‬‬‬‬‬‬‬‬‬‬‬‬‬‬‬‬‬‬‬‬‬‬‬‬‬‬‬‬‬‬‬‬‬‬‬‬‬‬‬‬‬‬‬‬‬‬‬‬‬‬‬‬‬‬‬‬‬‬‬‬‬‬‬‬‬‬‬‬‬‬‬‬‬‬‬‬‬‬‬‬‬‬‬‬‬‬‬‬‬‬‬‬‬‬‬‬‬‬‬‬‬‬‬‬‬‬‬‬‬‬‬‬‬‬‬‬‬‬‬‬‬‬‬‬‬‬‬‬‬‬‬‬‬‬‬‬‬‬‬‬‬‬‬‬‬‬‬‬‬‬‬‬‬‬‬‬‬‬‬‬‬‬‬‬‬‬‬‬‬‬‬‬‬‬‬‬‬‬‬‬‬‬‬‬‬‬‬‬‬‬‬‬‬‬‬‬‬‬‬‬‬‬‬‬‬‬‬‬‬‬‬‬‬‬‬‬‬‬‬‬‬‬‬‬‬‬‬‬‬‬‬‬‬‬‬‬‬‬‬‬‬‬‬‬‬‬‬‬‬‬‬‬‬‬‬‬‬‬‬‬‬‬‬‬‬‬‬‬‬‬‬‬‬‬‬‬‬‬‬‬‬‬‬‬‬‬‬‬‬‬‬‬‬‬‬‬‬‬‬‬‬‬‬‬‬‬‬‬‬‬‬‬‬‬‬‬‬‬‬‬‬‬‬‬‬‬‬‬‬‬‬‬‬‬‬‬‬‬‬‬‬‬‬‬‬‬‬‬‬‬‬‬‬‬‬‬‬‬‬‬‬‬‬‬‬‬‬‬‬‬‬‬‬‬‬‬‬‬‬‬‬‬‬‬‬‬‬‬‬‬‬‬‬‬‬‬‬‬‬‬‬‬‬‬‬‬‬‬‬‬‬‬‬‬‬‬‬‬‬‬‬‬‬‬‬‬‬‬‬‬‬‬‬‬‬‬‬‬‬‬‬‬‬‬‬‬‬‬‬‬‬‬‬‬‬‬‬‬‬‬‬‬‬‬‬‬‬‬‬‬‬‬‬‬‬‬‬‬‬‬‬‬‬‬‬‬‬‬‬‬‬‬‬‬‬‬‬‬‬‬‬‬‬‬‬‬‬‬‬‬‬‬‬‬‬‬‬‬‬‬‬‬‬‬‬‬‬‬‬‬‬‬‬‬‬‬‬‬‬‬‬‬‬‬‬‬‬‬‬‬‬‬‬‬‬‬‬‬‬‬‬‬‬‬‬‬‬‬‬‬‬‬‬‬‬‬‬‬‬‬‬‬‬‬‬‬‬‬‬‬‬‬‬‬‬‬‬‬‬‬‬‬‬‬‬‬‬‬‬‬‬‬‬‬‬‬‬‬‬‬‬‬‬‬‬‬‬‬‬‬‬‬‬‬‬‬‬‬‬‬‬‬‬‬‬‬‬‬‬‬‬‬‬‬‬‬‬‬‬‬‬‬‬‬‬‬‬‬‬‬‬‬‬‬‬‬‬‬‬‬‬‬‬‬‬‬‬‬‬‬‬‬‬‬‬‬‬‬‬‬‬‬‬‬‬‬‬‬‬‬‬‬‬‬‬‬‬‬‬‬‬‬‬‬‬‬‬‬‬‬‬‬‬‬‬‬‬‬‬‬‬‬‬‬‬‬‬‬‬‬‬‬‬‬‬‬‬‬‬‬‬‬‬‬‬‬‬‬‬‬‬‬‬‬‬‬‬‬‬‬‬‬‬‬‬‬‬‬‬‬‬‬‬‬‬‬‬‬‬‬‬‬‬‬‬‬‬‬‬‬‬‬‬‬‬‬‬‬‬‬‬‬‬‬‬‬‬‬‬‬‬‬‬‬‬‬‬‬‬‬‬‬‬‬‬‬‬‬‬‬‬‬‬‬‬‬‬‬‬‬‬‬‬‬‬‬‬‬‬‬‬‬‬‬‬‬‬‬‬‬‬‬‬‬‬‬‬‬‬‬‬‬‬‬‬‬‬‬‬‬‬‬‬‬‬‬‬‬‬‬‬‬‬‬‬‬‬‬‬‬‬‬‬‬‬‬‬‬‬‬‬‬‬‬‬‬‬‬‬‬‬‬‬‬‬‬‬‬‬‬‬‬‬‬‬‬‬‬‬‬‬‬‬‬‬‬‬‬‬‬‬‬‬‬‬‬‬‬‬‬‬‬‬‬‬‬‬‬‬‬‬‬‬‬‬‬‬‬‬‬‬‬‬‬‬‬‬‬‬‬‬‬‬‬‬‬‬‬‬‬‬‬‬‬‬‬‬‬‬‬‬‬‬‬‬‬‬‬‬‬‬‬‬‬‬‬‬‬‬‬‬‬‬‬‬‬‬‬‬‬‬‬‬‬‬‬‬‬‬‬‬‬‬‬‬‬‬‬‬‬‬‬‬‬‬‬‬‬‬‬‬‬‬‬‬‬‬‬‬‬‬‬‬‬‬‬‬‬‬‬‬‬‬‬‬‬‬‬‬‬‬‬‬‬‬‬‬‬‬‬‬‬‬‬‬‬‬‬‬‬‬‬‬‬‬‬‬‬‬‬‬‬‬‬‬‬‬‬‬‬‬‬‬‬‬‬‬‬‬‬‬‬‬‬‬‬‬‬‬‬‬‬‬‬‬‬‬‬‬‬‬‬‬‬‬‬‬‬‬‬‬‬‬‬‬‬‬‬‬‬‬‬‬‬‬‬‬‬‬Several limitations of this study should be noted. First, ‬as ‬the ‬data ‬are ‬cross-sectional, definitive statements regarding the causality of the included variables are not conclusive. Second, all of the included variables were measured with surveys (like the JCAT) that relied on perceptually based measures. Due to participants’ propensity to present a more favorable view of themselves due to social desirability, self-reporting questionnaires may introduce bias [38]. Socially desirable responses are most likely to occur to socially sensitive questions [39].‬‬‬‬‬‬‬‬‬‬‬‬‬‬‬‬‬‬‬‬‬‬‬‬‬‬‬ Nevertheless, results have shown important insights regarding the importance of respondent’s unique demographics as a potential in influencing the safety perceptions and provided the status of safety culture in a different view.‬‬‬‬‬‬ However, the study is a single site design with less number of respondents, and so the results are not generalizable.

## Conclusion

A different strategy to develop prospects for achieving and maintaining improvements in minimizing the repercussions of an error is through a strong just culture. This present study adds to the growing body of research that just culture principles help assess the commitment to patient safety by emphasizing some areas to be improved. It places paramount importance on encouraging voluntary error reporting and establishing a robust feedback system to address safety-related events. It also recognizes that errors present valuable opportunities for continuous improvement.

A strong measure of just culture is balancing individual’s accountability and perception of being supported to report safety incidents without the fear of consequences. In just culture, nurses perceive and feel more at ease taking the responsibility and accountability for their actions. While a no-blame practice gives an impression that their actions and errors may have no impact on patient safety. Although some dimension of just culture overlaps with the general safety culture, specific dimensions highlighted trust and balance accountability to be essentially improved.

## Implications for nursing management

The results of this study suggest fostering accountability and balance in practice rather than just a blame-free practice. To reduce potential for risky behaviors, leaders should train unit managers and supervisors on processes and policies that limit shortcuts and workarounds by their staff nurses. Additionally, the adoption of the just culture algorithm improves nurses’ perceptions of a fair and transparent approach to error investigation. Leaders must also employ the simple and accessible systems and ways for reporting errors. Leadership and management should encourage nurses to self-report mistakes and near-misses and offer prompt feedback on how errors were used for quality improvement.

## Data Availability

The datasets generated and/or analyzed during the current study are not publicly available due to the need to maintain the anonymity of participants and the confidentiality of the data. However, the datasets are available from the corresponding author on reasonable request.

## Abbreviations

AHRQ: Agency for Healthcare Research and Quality
HSOPSC: Hospital Survey on Patient Safety Culture
ISMP: Institute for Safe Medication Practices
JCAT: Just Culture Assessment Tool
SAQ: Safety Attitudes Questionnaire
SCQ: Safety Climate Questionnaire
